# Morphological diversity, phenolic acids, and antioxidant properties in eryngo (*Eryngium caucasicum* Trautv): Selection of superior populations for agri‐food industry

**DOI:** 10.1002/fsn3.2987

**Published:** 2022-07-22

**Authors:** Mehrnaz Hatami, Mahnaz Karimi, Ahmad Aghaee, Fatemeh Bovand, Mansour Ghorbanpour

**Affiliations:** ^1^ Department of Medicinal Plants, Faculty of Agriculture and Natural Resources Arak University Arak Iran; ^2^ Department of Horticultural Sciences Sari Agricultural Sciences and Natural Resources University Sari Iran; ^3^ Department of Biology, Faculty of Science University of Maragheh Maragheh Iran; ^4^ Department of Agronomy and Plant Breeding Islamic Azad University Arak Iran

**Keywords:** chicoric acid, DPPH, eryngo, flavonoids, phenotypic variability, rosmarinic acid

## Abstract

Eryngo (*Eryngium caucasicum* Trautv) a widespread species of the Apiaceae reveals high nutritional value and therapeutic properties due to the significant content of biologically active metabolites such as essential oils, phenolic compounds, and flavonoids. The present study was performed to evaluate the morphological and biochemical variability and antioxidant properties of naturally grown populations of eryngo. One‐way ANOVA showed significant (*p* < .01) differences in the majority of parameters measured among the studied populations. The range of fresh weight was from 1.3 to 12.0 g/plant, while dry weight varied from 0.01 to 6.0 g/plant. The highest variation was observed for essential oil yield (CV = 205.32%) followed by essential oil content (CV = 126.23%) and chicoric acid content (CV = 71.18%). Total phenolics content varied from 8.85 to 88.15 mg GAE/g extract. Total flavonoids value ranged from 5.41 to 134.40 mg QE/g extract. Rosmarinic acid and chicoric acid contents varied from 0.118–1.234 and 0.014–0.597 μg/g DW, respectively. DPPH free radical scavenging activity varied from 76.12 to 513.5 μg/mL, while it ranged from 156.7 to 477.1 μg/mL with the ferrous ions (Fe^2+^) chelating assay. Rosmarinic acid and chicoric acid showed a significant and positive correlation (*r*_0.01_ = 0.81 and *r*_0.05_ = 0.40) with total phenolics, respectively. The Ward dendrogram analysis revealed two different clusters based on the parameters measured, confirming high morpho‐phytochemical variability among the individuals and populations. Principal component analysis (PCA) revealed eight PCs which contributed to 99.97% of the overall variance, and leaf length, essential oil content, and antioxidant activity in terms of DPPH and Fe^2+^ chelating techniques were the most effective attributes for characterizing and selecting the studied population. Based on the traits related to vegetative yield and antioxidant properties, eight individuals from two populations were superior for breeding and/or farming programs.

## Graphical abstract

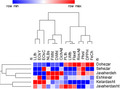

## 1 INTRODUCTION

The genus *Eryngium* L. with more than 250 species is the largest in the family Umbelliferae (Apiaceae). It is broadly distributed throughout temperate areas of every continent. The *Eryngium* spp. are naturally grow in Central Asia, North and South America, North Africa, Australia, and Eurasia (Pimenov and Leonov, 1993; Calviño et al., 2008; Cortés‐Fernández et al., 2022). In the wild flora of Iran, there are nine native species of *Eryngium* (Ghahreman 1997), of which eryngo (*Eryngium caucasicum* Trautv) is one of the most common and widespread species. It is a perennial herbaceous plant that has been distributed in Caucasia and North Eastern Anatolia across Northern, Middle Asia, Southern Russia, Eastern and Central Iran, Afghanistan, Pakistan, and Western Himalaya (Khoshbakht et al., 2007; Wörz 2004; Behmanesh et al., 2019). Due to its constantly increasing demand, *E. caucasicum* was introduced as a new crop plant in household gardens around the Caspian Sea and Alborz mountains in northern Iran.

Like the other plants of this family, *Eryngium* has been widely used as food, ornamental, vegetable, and/or in traditional medicine locally or worldwide (Zhang et al., 2008). The shoot parts of *E. caucasicum* are often used as a cooked vegetable, and/or as an aroma additive in the preparation of many traditional foods (Khoshbakht et al., 2007). Its fragrant young leaves are used as a stuffing mix in fish and chicken, and the cooked leaves are also consumed in soup or with yogurt (Eslami et al., 2011).

In herbal medicine, infusions of shoot and root tissues of the various *Eryngium* spp. have been used as diuretic, emmenagogue, spasmolytic, aphrodisiac, appetizer, antitussive, and stimulant (Duke, 2002). Moreover, *Eryngium* extracts and the isolated constituents have exhibited in vitro cytotoxicity impacts on various human tumor cell lines, anti‐flatulence, anti‐snake, anti‐inflammatory, anti‐hyperglycemic and scorpion venoms, antibacterial, antifungal, antimalarial, and antioxidant properties (Kartal et al., 2005; Khoshbakht et al., 2007; Ebrahimzadeh et al., 2009; Wang et al., 2012; Kowalczyk et al., 2014).

The chemical composition of *E. caucasicum* mainly consists of phenols, flavonoids, coumarins, and essential oils, presenting various redox capacities and pharmacological properties (Hashemabadi et al., 2010; Eslami et al., 2011; Behmanesh et al., 2019). Among the various plant secondary metabolites, phenolic compounds are of the most varied groups that have important health benefits, as they are believed to be the main factor for reducing cardiovascular disease risk in communities that use phenolics‐rich foods and dietary supplements (Leopoldini et al., 2011; Sukhadiya et al., 2021). In addition, they play a vital role in plant adaptation/and or acclimation to various environmental perturbations.

Plant growth‐related parameters, secondary metabolites biosynthesis, and their biological properties are influenced by environmental stresses, growth stimulants, and geographical and genetic factors (Sampaio et al., 2016; Ghorbanpour et al., 2016; Selmar et al., 2017; Meftahizadeh et al., 2019; Pourhosseini et al., 2020; Mohammadi et al., 2021; Mirheidari et al., 2022). Previous studies have shown the importance of certain morphological and biochemical traits regarding the productivity of plants in the Apiaceae family (Ninou et al., 2017; Adelifar et al., 2020; Jorkesh et al., 2020;). However, little is known about the phenotypic diversity and biochemical variation of *E. caucasicum* in different natural habitats. Thus, this study aimed to evaluate the morphological diversity and biochemical variation as well as antioxidant capacity in *E. caucasicum* populations from the northern parts of Iran.

## 2 MATERIALS AND METHODS

### 2.1 Plant materials and collection sites

**FIGURE 1:**
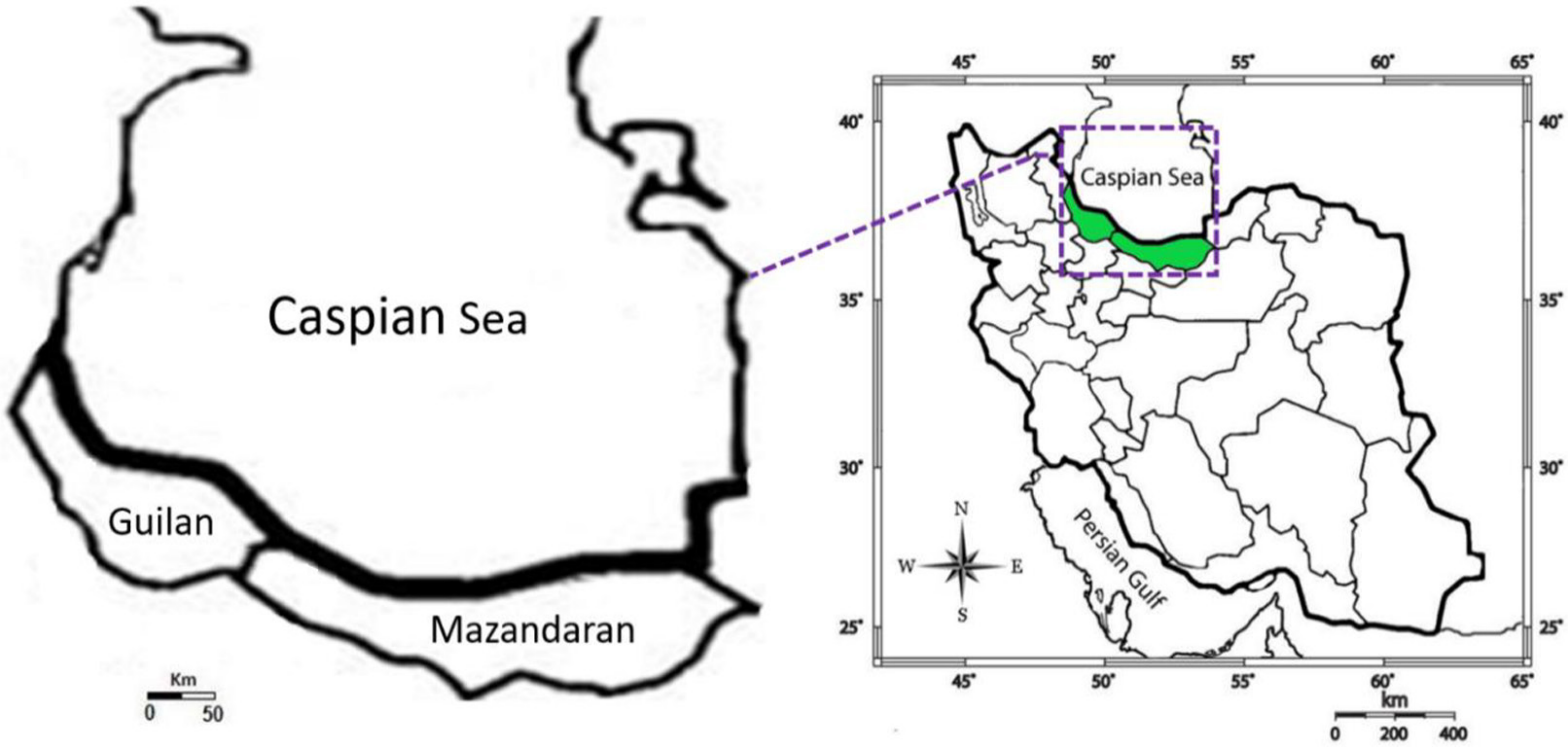
Map of the collection areas, Mazandaran (Dohezar, Sehezar, Javaherdeh, and Kelardasht) and Guilan (Eshkevar and Javaherdasht) for the studied populations of eryngo (*Eryngium caucasicum* Trautv) from north of Iran (the geographic and climatic characteristics of these locations are visible in Table 1)

**FIGURE 2:**
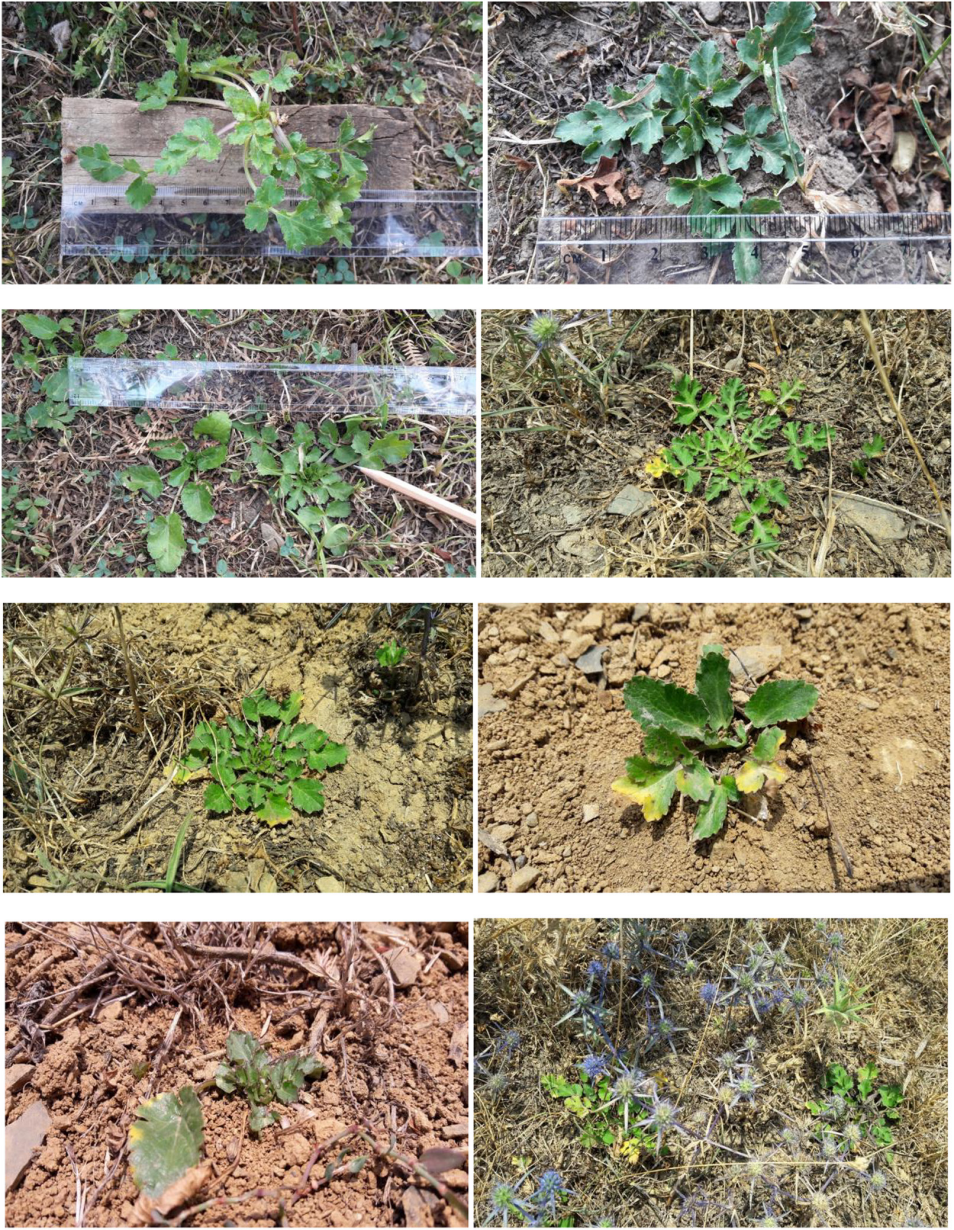
Variability of morphological traits in eryngo (*Eryngium caucasicum* Trautv) populations studied

In the present study, 60 naturally grown individuals of eryngo (*Eryngium caucasicum* Trautv) were sampled from different habitats/populations of the Mazandaran (Javaherdeh, Dohezar, Sehezar, and Kelardasht) and Guilan (Javaherdashte and Eshkevar) provinces in the north of Iran. The distribution map of the studied populations is shown in Figure 1, and the climatic and geographical characteristics corresponding to these areas are presented in Table 1. The aerial parts/leaf materials were harvested during the growing season July–August 2019. Figure 2

**TABLE 1:** Geographic and climatic characteristics of collection sites for the eryngo (*Eryngium caucasicum* Trautv) populations studied

	Population					
	Mazandaran‐Dohezar	Mazandaran ‐Sehezar	Mazandaran‐ Javaherdeh	Mazandaran ‐ Kelardasht	Guilan ‐Eshkevar	Guilan‐Javaherdasht
Characteristic						
Latitude (N)	50°50′52.077”	50°51′3.201”	50°28′22.438”	51°12′5.427”	50°4′ 2.736”	37°09′57”
Longitude (E)	36°42′39.433″	36°43′52.744”	36°51′20.856”	36°29′54.951”	36°52′10.546”	49°52′25”
Altitude (m)	1828	1688	1361	1180	910	1030
Mean annual air temperature (°C)	9.1	10.4	12.5	15.3	13.8	11.6
Mean annual rainfall (mm)	564	638	980	450	820	730

### 2.2 Morphological analysis

The key morphological parameters such as leaf size (length and width) and petiole length were determined using a digital caliper. The fresh and dry weights of aerial parts were measured using a precision electronic balance in the range 0.001–1 g. In addition, the number of leaves/branches per plant was recorded manually. For these parameters, data were collected from 10 randomly selected individual plants in each population and their mean values were considered for statistical analysis.

### 2.3 Biochemical analysis

The shoot parts of all collected samples were air‐dried in the shade at ambient temperature (22–25°C) until constant weight and were considered for further laboratory/biochemical analyses.

#### 2.3.1 Essential oils isolation

For essential oils isolation, 30 g of the coarsely ground plant materials was hydrodistillated in a Clevenger‐type apparatus using 500 ml of distilled water for approximately 3 h per sample based on the method quoted in British Pharmacopoeia (1998). After measuring the essential oil content (expressed as percentage w/w), the essential oil yield was calculated as dry weight × essential oil content of the sample (expressed as g/plant) (Singh et al., 2013).

#### 2.3.2 Measurement of total phenolics and flavonoids

Total phenolic content of the collected samples was measured using the Folin–Ciocalteu reagent with reference/standard compound, gallic acid (GA), following the procedure of Meda et al. (2005), and the final data were expressed as milligram of GAE per gram of dried extract.

The spectrophotometric assay was used to determine total flavonoid content based on aluminum chloride with quercetin (Q) as standard following the procedure of Chang et al. (2002). The obtained data were expressed as milligrams of QE per gram of dried extract.

#### 2.3.3 HPLC analysis for identification of phenolic compounds

In order to identify phenolic compounds of *E. caucasicum* samples, the methanolic extract was analyzed by ELISA, PDA, and MASS detectors of Shimadzu Corporation‐LCMS‐8060.According to the method of Chen et al. (2019), the contents of rosmarinic acid and chicoric acid were calculated by high‐performance liquid chromatography (HPLC). Briefly, in a conical flask, 0.5 g of dried *E. caucasicum* powder was weighed and mixed with 10 ml of 80% methanol solution, followed by sonication at 40°C for 60 min. After initial centrifugation at 12,000 *g* for 1 h, the supernatants were filtered via 0.22 μm PEs membrane filter, and the filtrate was used as the test solution. Then, 10 μl of test solution was injected into an U.S. Waters Alliance 2695 HPLC system, equipped with the Uv 2487 Dual ʎ Absorbance Detector and the C_18_ column, with dimensions of 4.6 mm × 150 mm and 3.5 mm, with Millennium 32 software for analysis on an HPLC chromatography.

### 2.4 Antioxidant activity

#### 2.4.1 DPPH scavenging assay

The antiradical activity of the collected samples was assessed based on DPPH radical scavenging activity following the protocol explained by Brand‐Williams et al. (1995). The scavenging activity of DPPH was estimated through the following equation (Okoh et al., 2014).

$\text{DPPH activity}\;\left(\%\right)=\left[\frac{\mathrm{Abs}\;\text{control}-\mathrm{Abs}\;\text{sample}}{\mathrm{Abs}\;\text{control}}\right]\times 100$

#### 2.4.2 Ferrous iron (Fe^2+^) chelating assay

The Fe^2+^ chelating property was assayed according to the method of Zhengjun et al. (2008).

### 2.5 Statistical data analysis

Data were subjected to a one‐way ANOVA test using SAS (SAS Institute,). The normality of all data was preliminary checked using the Shapiro–Wilk normality test. Relationships among the individuals/and populations were evaluated by principal component analysis (PCA) using SPSS® software. Cluster analysis (UPGMA) was performed by the Euclidean distance method using PAST software. Antioxidant measurements were done in triplicate

## 3 RESULTS AND DISCUSSION

### 3.1 Morphological variations

One‐way ANOVA results showed a statistically‐significant (*p* < .01) difference in all traits measured among the studied populations. Therefore, it was possible to select the *E. caucasicum* populations for different values of a trait. The highest variation was observed for plant dry weight (CV = 81.41%) followed by leaf width (CV = 66.60%) and petiole length (CV = 52.17%). In contrast, the number of leaves/branches per plant and leaf length showed the lowest CVs, 38.75% and 42.13%, respectively (Table 2).

**TABLE 2:** Descriptive statistics for the morphological and biochemical traits and antioxidant activities in the studied eryngo (*Eryngium caucasicum* Trautv) populations

No.	Trait	Abbreviation	Unit	Min	Max	Mean	SD	CV (%)
1	Leaf length	LLth	mm	1.0	42.0	21.5	9.08	42.13
2	Leaf width	LWth	mm	1.0	18.0	6. 5	4.36	66.60
3	Petiole length	PLth	mm	12.0	96.0	43.1	22.48	52.17
4	Number of leaf/branches	NLBs	No.	1.0	3.0	1.78	0.691	38.75
5	Plant fresh weight	FWht	g	1.3	12.0	5.34	2.772	51.86
6	Plant dry weight	DWht	g	0.01	6.0	1.93	1.573	81.41
7	Essential oil content	EOsC	%	0.011	0.30	0.028	0.036	126.23
8	Essential oil yield	EOsY	g/plant	0.0001	1.14	0.073	0.015	205.32
9	Rosmarinic acid	RosAd	μg/g DW	0.118	1.234	0.494	0.251	50.81
10	Chicoric acid	CichAd	μg/g DW	0.014	0.597	0.226	0.161	71.18
11	Total phenolics	Phnl	mg GAE/g extract	8.85	88.15	35.96	19.65	54.64
12	Total flavonoids	Flavd	mg QE/g extract	5.41	134.4	45.34	31.06	68.49
13	2,2‐diphenyl‐1‐picrylhydrazyl	DPPH	μg/mL	76.12	513.5	167.01	72.48	43.40
14	Ferrous ions (Fe^2+^) chelation	FeCh	μg/mL	156.7	477.1	249.45	57.10	22.89

Abbreviations: CV, Coefficient of variation; DW, dry weight; GAE, Gallic acid; QE, Quercetin; SD, Standard deviation.

The most important traits of eryngo in food industries and various local foods are related to leaf growth and vegetative yield (Khoshbakht et al., 2007). Due to the photosynthesis process and subsequent secondary metabolites biosynthesis, leaf dimension and traits involving biomass are the most important parameters to be considered in agronomy and breeding/genomics programs (Ievinsh et al., 2020; Li et al., 2021).

Here, leaf length varied from 1.0 to 42.0 mm and leaf width ranged from 1.0 to 18.0 mm. Furthermore, petiole length varied from 12.0 to 96.0 mm, and the number of leaves/branches ranged from 1.0 to 3.0 (Table 2). The range of fresh weight was from 1.3 to 12 g per plant, while dry weight varied from 0.01 to 6 g per plant with an average of 1.93 g/plant. The majority of individuals within the populations showed medium plant dry yield (35 individuals, 58.30%) followed by a high dry matter (5 individuals, 12.00%). Little is known about the growth habitat, phenotypic diversity, and biomass variation of eryngo species worldwide to compare with our findings. However, significant variation has been reported in complexity (total number of shoots per individual), the number of leaves and flowers, and various developmental phase transitions (vegetative to generative and vice versa) of *E. maritimum* from Southeastern Baltic coast (N56°48′4″, E21°4′4″) (Ievinsh et al., 2020). In a study on the diversity of morphological and biochemical traits of 52 froriepia (*Froriepia subpinnata* Ledeb. Bail, Apiaceae) accessions from Guilan province (Iran), Jorkesh et al. (2020) reported that leaf and leaflet lengths were the most divers among various parameters, so that the highest variation was observed in leaflet width and leaf number. It has been acknowledged that plant morpho‐physiological parameters, chemical constituents, and biological activities are influenced by both environmental and genetic factors (Heywood, 2002). In particular, it was established that photochemical performance of photosynthesis in *E. maritimum* is extremely sensitive to periods of increased precipitation (Andersone et al. 2011). Also, prolonged periods of precipitation together with low air temperature were devastating for eryngium seeds after ripening, resulting in extremely poor seed germination rate and subsequent seedling growth (Necajeva and Ievinsh 2013). In the present study, the greater biomass accumulation of *E. caucasicum* from Javaherdeh and Eshkevar sites may be attributed to the higher mean annual rainfall and air temperature compared to the other studied locations (Table 1). In addition, the highest leaf length ofthe Kelardasht population could be linked to the site effect because this region is closed to Hyrcanian forests and Sard‐ Abroud River along with longer humidity retention. However, plant species trigger various physiological and biochemical mechanisms to cope in situations of growth‐limiting resources and environmental perturbations (Bermúdez and Retuerto 2014). The pictures of aerial parts of the studied populations of *E. caucasicum* plants are shown in Figure 1.

There were significant (positive/and or negative) correlations between some traits as presented with Pearson's correlation general overview and coefficients (Table 3). Leaf length showed a significant and positive correlation with leaf width (*r*_0.05_ = 0.32), petiole length (*r*_0.01_ = 0.48), number of branches (*r*_0.05_ = 0.31), plant fresh weight (*r*_0.01_ = 0.61), and dry weight (*r*_0.01_ = 0.50), in consistent with the previous findings in *F. subpinnata* (Jorkesh et al., 2020). In contrast, Ievinsh et al. (2020) found a negative correlation between the number of leaves per individual and the number of vegetative shoots per individual in *E. maritimum*.

**TABLE 3:**
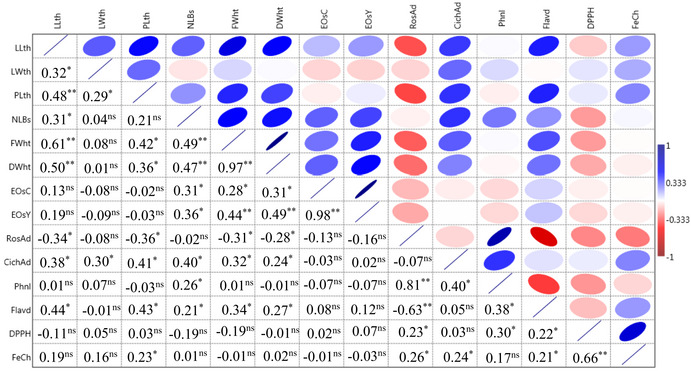
Pearson's correlation general overview (upper triangle) and coefficients (lower triangle) between the morphological and biochemical traits and antioxidant activities in the studied eryngo (*Eryngium caucasicum* Trautv) populations. Colors in the matrix boxes show the strength and direction of the relationships: intense red and blue colors display strong negative and positive correlations, respectively

ns, *, and **: non‐significant, and significantly different at 5 and 1% probability levels, respectively. For the explanation of traits abbreviation, please see Table 2.

The PCA is a statistical technique that reduces the dimensionality of large datasets, summarizes information content, and interprets the relationship between variables. Here, PCA was used to better understand the relationships among the studied populations. The PCA revealed that the first eight components (PC1–PC8) accounted for 99.97% of the overall variance (Table 4). Values greater than or equal to 0.50 were considered to be significant for a specific trait. The PC1 explained 72.74% of the total variance represented by leaf length (5.04) with positive correlations. Other morphological parameters including plant fresh weight, plant dry weight, and the number of leaf/branches per plant were placed into the PC5, representing 0.09% of the total variance. The PC6 and PC7 explained 0.03% and 0.004% of the total variance detected and revealed positive correlations with petiole length and leaf width, respectively (Table 4). In a study on phenotypic diversity of Greek dill (*Anethum graveolens* L., Apiaceae) landraces, Ninou et al. (2017) reported that the PCA reduced the 26 agro‐morphological traits to eight dimensions which represented the 83.4% of the total variability.

**TABLE 4:** Eigenvectors of principal component axes from PCA for the morphological and biochemical traits and antioxidant activities in the studied eryngo (*Eryngium caucasicum* Trautv) populations. For the explanation of traits abbreviation, please see Table 2

Trait	Component							
	1	2	3	4	5	6	7	8
LLth	5.04**	0.01	0.003	0.01	0.14	0.03	0.91**	0.17
LWth	0.0004	0.001	−0.002	0.0001	0.01	0.07	0.58**	−0.05
PLth	0.0031	0.02	0.01	0.03	0.32	0.93**	−0.1	0.01
NLBs	−0.001	0.004	−0.001	0.01	0.80**	−0.05	−0.1	0.96**
FWht	−0.004	0.02	0.01	0.03	0.81**	−0.27	0.01	−0.07
DWht	−0.002	0.009	0.007	0.01	0.56**	−0.18	−0.2	−0.1
EOsC	−9.36**	5.13**	9.5**	−6.3**	0.002	−0.004	−0.002	0.01
EOsY	−0.0001	0.0003	0.0004	−0.0002	0.01	−0.02	−0.02	0.05
RosAd	−0.0008	−0.002	−0.006	0.005	−0.01	−0.009	−0.02	−0.03
CichAd	0.0002	0.0008	−0.001	0.003	0.01	0.01	0.03	0.05
Phnl	−0.04	−0.04	−0.49	0.86**	−0.02	−0.01	−0.001	−0.01
Flavd	−0.003	0.56**	0.70**	0.43	−0.04	−0.02	−0.003	−0.005
DPPH	0.81**	−0.47	0.27	0.17	0.004	−0.003	0.001	0.0006
FeCh	0.57**	0.47	−0.42	−0.18	−0.002	−0.003	−0.003	−0.001
Eigenvalue	7189.9	1648.1	803.9	227.6	9.5	3.2	0.46	0.30
% of variance	72.74	16.67	8.13	2.30	0.09	0.03	0.004	0.003
Cumulative %	72.74	89.41	97.54	99.84	99.93	99.96	99.964	99.967

**Eigenvalues ≥0.50 are significant at *p* = 0.01 probability level.

**FIGURE 3:**
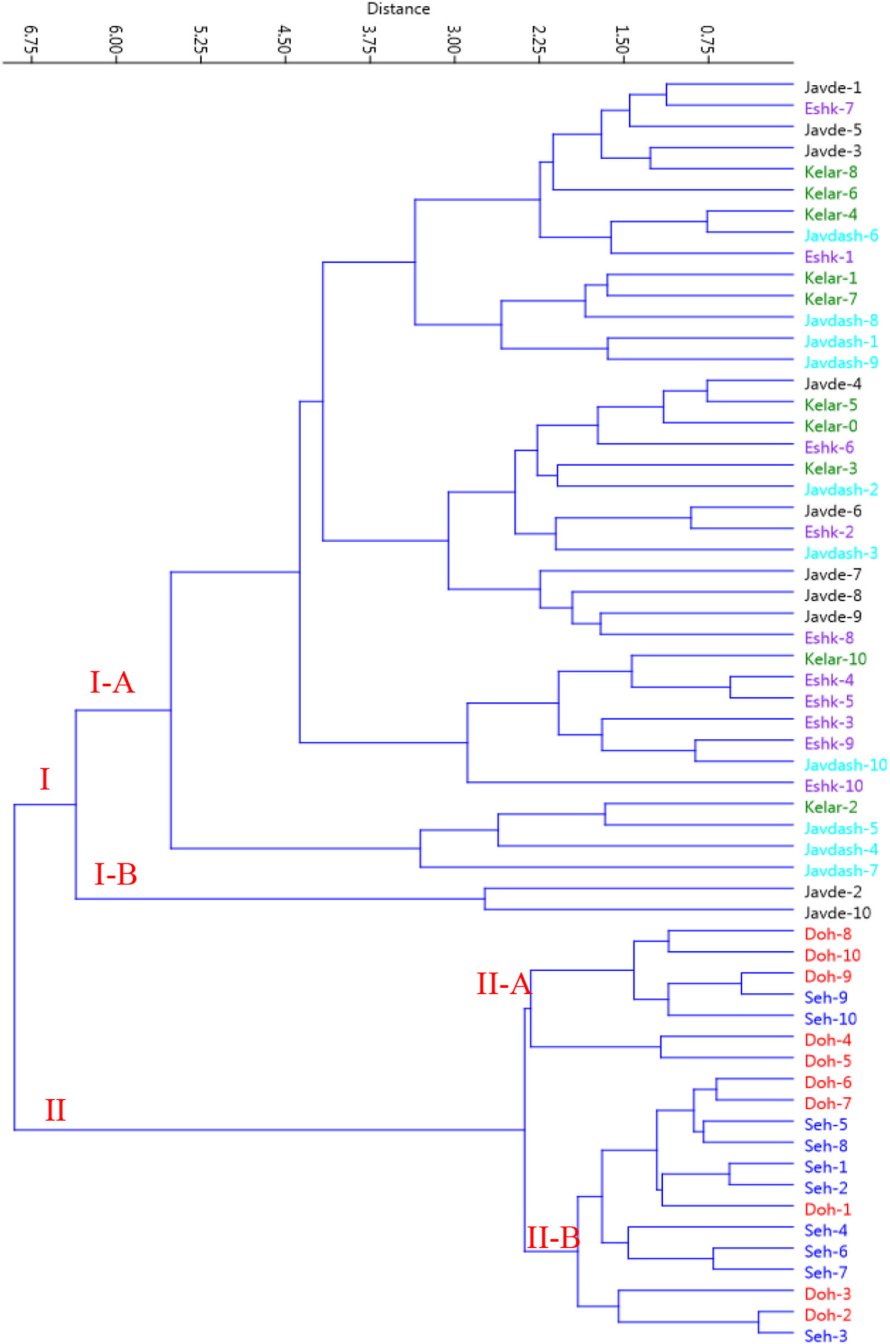
Ward cluster analysis of the studied eryngo (*Eryngium caucasicum* Trautv) populations (Javaherdeh, Kelardasht, Dohezar, Sehezar, Javaherdasht, and Eshkevar represented by black, green, red, blue, aqua, and blue‐violet color symbols, respectively) based on the morphological traits by Euclidean distances

**FIGURE 4:**
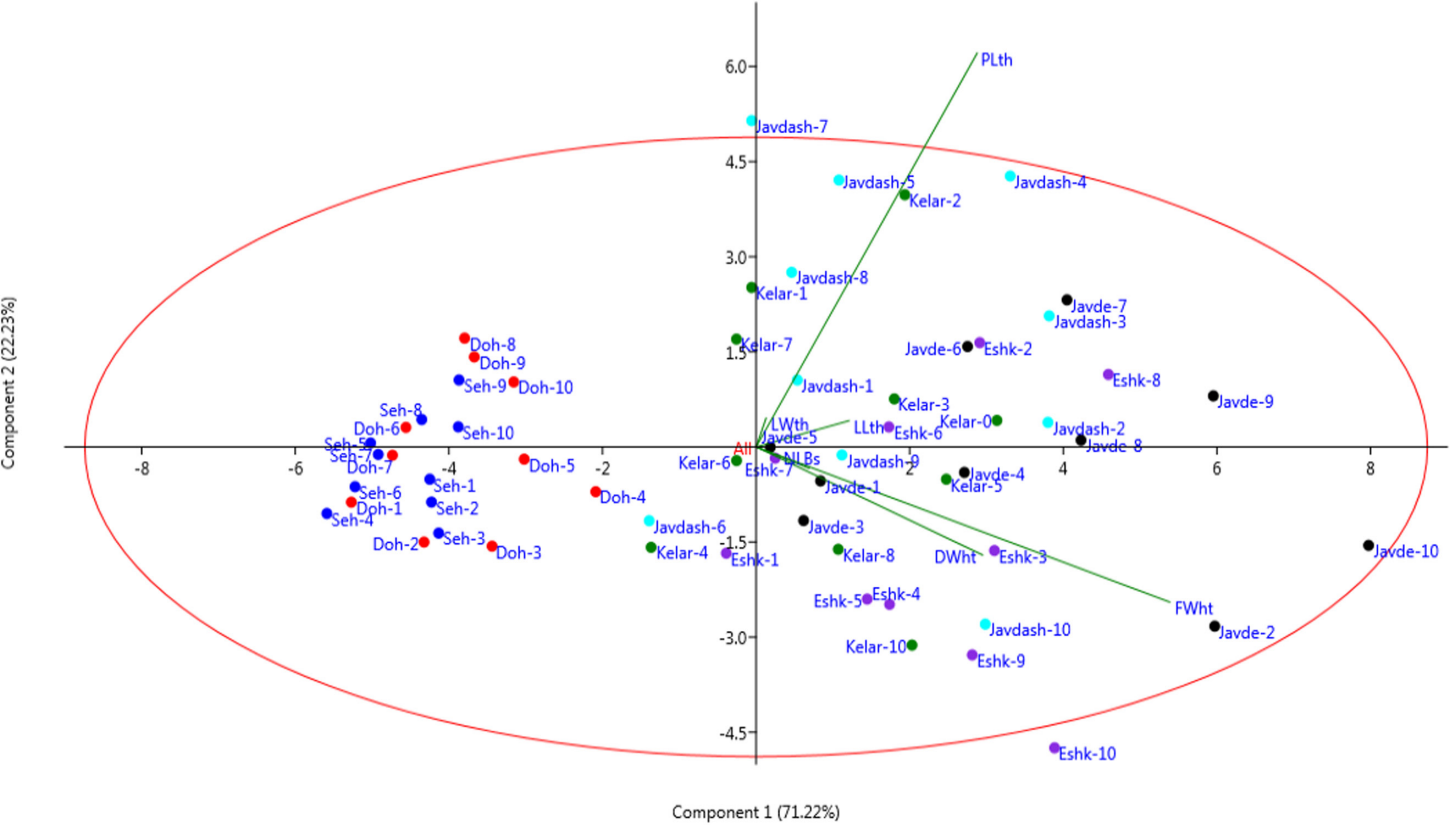
Two‐dimensional scatter plot for PC1 and PC2 based on the morphological traits for the individuals (No.: 1–10) of eryngo (*Eryngium caucasicum* Trautv) populations (Javaherdeh, Kelardasht, Dohezar, Sehezar, Javaherdasht, and Eshkevar, represented, by black, green, red, blue, aqua, and blue‐violet color symbols, respectively)

The cluster analysis based on the morphological traits performed by Euclidean distance using Ward method (Figure 3) classified the individuals of all populations into two major clusters of I and II. The first cluster (I) was grouped into two subclusters (I‐A and I‐B). Subcluster I‐A comprised 38 individuals, and subcluster I‐B consisted tw2o individuals. The second cluster (II) included 20 individuals forming two subclusters, II‐A and II‐B, which consisted of 7 and 13 individuals, respectively. There was high morphological diversity (mainly characterized by higher values in petiole length, leaf length, and plant fresh and dry weights) among the individuals (Figure 4). Jawdat et al. (2010) in a comprehensive morphological, molecular, and geographical study regarding *Eryngium* L. spp. in Syria showed the high adaptation potentials of *E. creticum* and *E. desretorum* spp. to various environments (mountains, semidesert, coastal sandy beaches, and saline conditions), causing to increase in their diversity.

Furthermore, the scatter plot designed in terms of PC1 and PC2 (Figure 4) showed morphological variations among individuals in studied populations. As shown, the individuals with close proximity were more similar based on the impressive parameters in PC1 and PC2 and, therefore, were placed in the same group. The loading plot for PC1 and PC2 pointed out the importance of petiole length, leaf length, and biomass accumulation for interpreting the observed variance among individuals. Here, the variability observed among the populations (which appeared as their clear grouping in the scatter plot and UPGMA analyses) could be linked with the population size, distance between the populations, and geographical locations (Minasiewicz et al., 2011). These could be especially pronounced in clonally propagating plants like *E. caucasicum*.

### 3.2 Biochemical and antioxidant evaluations

According to the ANOVA, significant (*p* < .01) variations were observed among the examined individuals/and populations in terms of the biochemical traits and antioxidant activities measured. The highest variation was observed for essential oil yield (CV = 205.32%), followed by essential oil content (CV = 126.23%) and chicoric acid content (CV = 71.18%). In contrast, antioxidant activity in terms of ferrous ions (Fe^2+^) chelating power and DPPH showed the lowest CVs, 22.89 and 43.40%, respectively (Table 2). Moreover, the rest biochemical parameters (rosmarinic acid, total phenolics, and flavonoid contents) showed CVs greater than 50.00%, displaying a high diversity among the studied populations.

Essential oils content varied from 0.011% to 0.30% with an average of 0.28% (Table 2). These values have been in accordance with the previous data reported was as 0.29% in *E. amethystinum* from Amiata Mount, Italy (Flamini et al., 2008), and 0.06%–0.13% in *E. maritimum* from Corsica and Sardinia (Darriet et al., 2014). The essential oils yield ranged from 0.0001 to 1.14 g/plant (Table 2). Volatiles of the essential oils are largely responsible for the taste of foods. Essential oils of the *Eryngium* species, even if used in small amounts, its pungent unique odor gives the characteristic flavor to the dishes in which it is incorporated (Flamini et al., 2008). The differences in essential oils content and composition of various *Eryngium* species and even within the same species may be ascribed to the characteristics of the growth regions (Cardozo et al., 2004; Sefidkon et al., 2004). All these characteristics should be taken into consideration when the plant is used as an aroma source.

**FIGURE 5:**
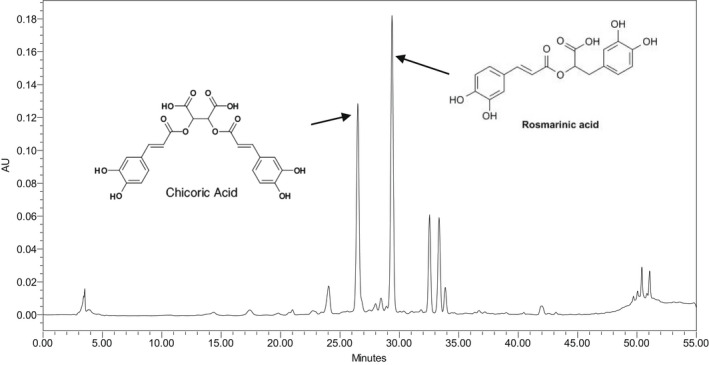
HPLC chromatogram of phenolic acids (rosmarinic acid and chicoric acid) fractions from leaves of eryngo (*Eryngium caucasicum* Trautv)

Rosmarinic acid content varied from 0.118 to 1.234 μg/g DW with an average of 0.494 μg/g DW (Table 2). In our experiment, a higher content (>0.40 μg/g DW) of this hydroxylated polyphenol was found in most of the individuals and agreed with Kikowska et al. (2022), who reported that rosmarinic acid was the predominant phenolic acid with the highest content in all of the examined tissues of *Eryngium* spp. in vitro. Also, rosmarinic acid was previously found most notably in other *Eryngium* species such as *E. campestre* (Kikowska et al., 2016), *E. maritimum* (Kikowska et al., 2014), and *E. planum* (Thiem et al., 2013). The range of chicoric acid content was from 0.014 to 0.597 μg/g DW (Table 2). The major phenolic acids in *E. serbicum* were reported as chlorogenic and rosmarinic acid (Vukic et al. (2018). In a study by Le Claire et al. (2005), rosmarinic acid was identified in *E. maritimum*, *E. amethystinum*, *E. campestre,* and *E. alpinum*. In contrast, rosmarinic acid was not detected in the analyzed samples of *E. amethystinum* (Kremer et al., 2021). The difference might be attributed to ecological situations, edaphic factors, and genetic variations inside the species. HPLC chromatogram of rosmarinic acid and chicoric acid fractions in the leaves of *E. caucasicum* collected from Javaherdeh is shown in Figure 5.

Total phenolic content varied from 8.85 to 88.15 mg GAE/g extract. Total flavonoid content ranged from 5.41 to 134.40 mg QE/g extract (Table 2). Phenolic acids and flavonoids, precious bioactive secondary metabolites of *Eryngium* spp., serve a vital role in plants metabolism; their effect, mainly as antioxidants as well as folklore remedies, on human health has been of intense interest to researchers in recent years (Kikowska et al., 2019). Previous studies have revealed the high amounts of total phenolic compounds in different extracts of *Eryngium* spp., for example, in *E. campestre* (Guneş et al., 2014; Bouzidi et al., 2017). In addition, the total phenolics and flavonoids content of aqueous, *n*‐hexan, and ethyl acetate extracts in *E. caucasicum* were reported as 214.18, 29.06, and 140.57 mg GAE/g extract, and 75.36, 97.37, and 31.51 mg/QE g extract, respectively (Nabavi et al., 2012).

In the current study, the antioxidant capacity of the methanol extract of *E. caucasicum* determined using the free radical DPPH scavenging technique varied from 76.12 to 513.5 μg/mL, while it ranged from 156.7 to 477.1 μg/mL with the ferrous ions (Fe^2+^) chelation assay (Table 2). Moreover, samples collected from the Dohezar region exhibited the highest DPPH scavenging activity and chelating power as compared to the other studied populations. Antioxidant activity of ethanol extracts of the aerial and roots parts of *E. campestre* from Kosovo has previously been evaluated using various testing systems, and findings suggest that, in the DPPH assay, root extract revealed higher radical scavenging activity compared to the extract of the shoot (Nebija et al. 2009). In a study by Ebrahimzadeh et al. (2009), antioxidant activities of methanol extracts of leaves and inflorescence of *E. caucasicum* were investigated using six *in vitro* assay systems, and results showed that leave extract exhibited better Fe^2+^ chelating ability than EDTA. Antioxidant molecules and phenolic compounds of plant origin are important to food and pharmaceutical industries due to their unique taste, flavor, and health‐promoting properties. The antioxidant activity of phenolic constituents and flavonoids is basically related to the phenolic rings and hydroxyl groups that enable them to scavenge free radicals, chelate metal ions, and inhibit lipoxygenase activity (Minatel et al., 2017). Here, the antioxidant activity of *E. caucasicum* might be attributed to their phenolic compounds; rosmarinic acid and chicoric acid were detected in leaf methanol extract (Figure 5).

The correlation coefficients clearly measure the relationship between two parameters. Statistically significant correlations were found between some traits measured (Table 3). As shown, essential oil yield was significantly and positively correlated with essential oil content (*r*_0.01_ = 0.98), plant dry weight (*r*_0.01_ = 0.49), plant fresh weight (*r*_0.01_ = 0.44), and the number of branches per plant (*r*_0.05_ = 0.36). Rosmarinic acid and chicoric acid content showed a positive and significant correlation with total phenolics, *r*_0.01_ = 0.81 and *r*_0.05_ = 0.40, respectively. Total phenolic content was significantly and positively correlated with total flavonoids (*r*_0.05_ = 0.38) and radical scavenging activity (*r*_0.05_ = 0.30) and agreed with the previous reports in *Eryngium* spp. (Rjeibi et al., 2017; Kikowska, et al., 2022). Antioxidant activity based on DPPH was positively and significantly (*r*_0.05_ = 0.66) correlated with antioxidant activity results obtained with Fe^2+^ chelating assay. The correlation between total phenol content and antioxidant activity has previously been reported by many authors and their results showed a statistically significant correlation between phenol content and antioxidant potential (Johari & Khong  2019; Guo et al., 2021). In addition, the antioxidant activity of essential oils cannot be ignored either (Farias et al., 2020).

The PC1 explained 72.74% of the total variance was represented by essential oil content (9.36), DPPH (0.81), and ferrous ions (Fe^2+^) chelation (0.57) with significant correlations (Table 4). The PC2 included two traits including essential oil content (5.13) and total flavonoids (0.56), accounting for 16.67% of the total variance. Total phenolics represented positive and significant correlations with PC4 (0.86).

**FIGURE 6:**
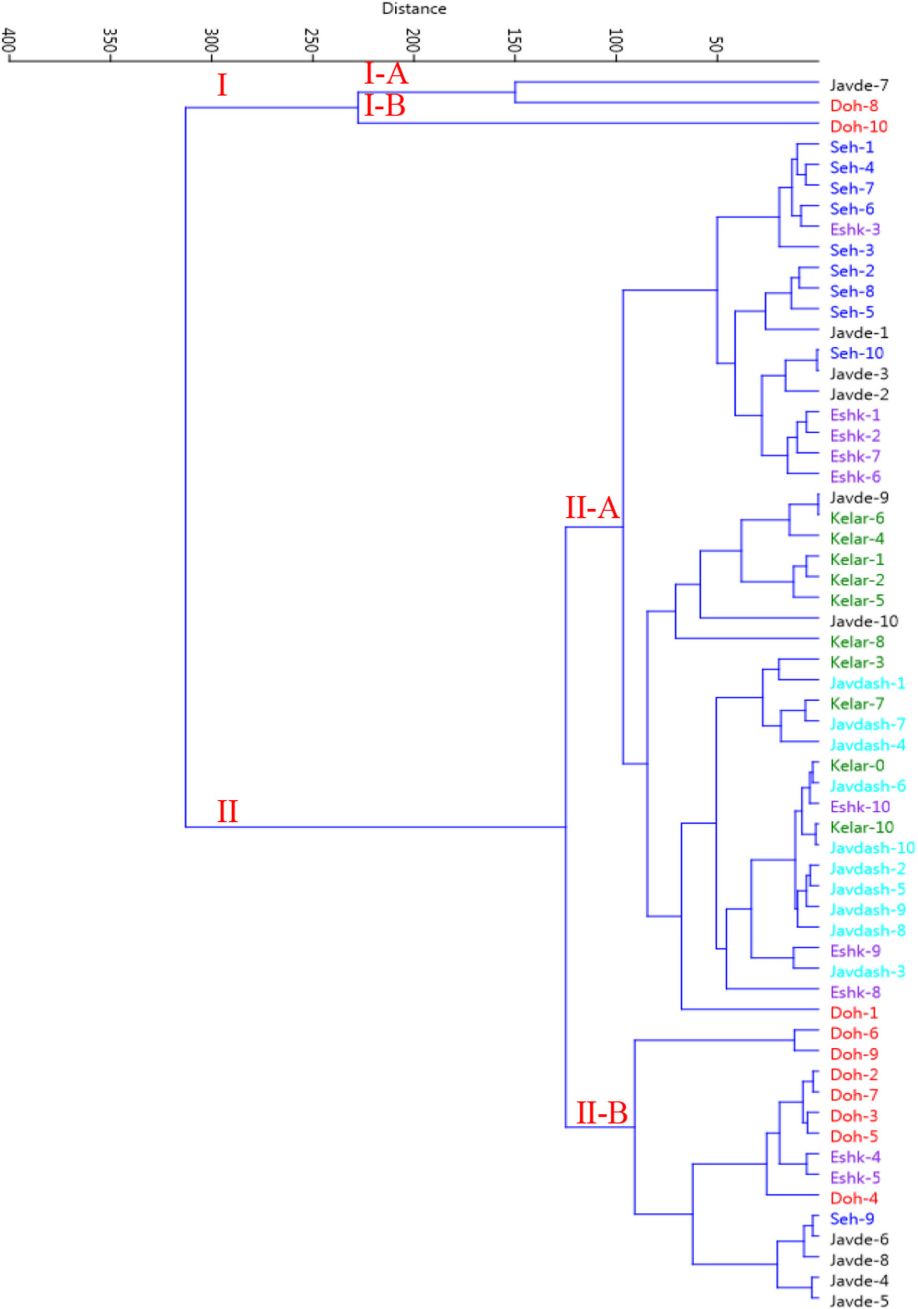
Ward cluster analysis of the studied eryngo (*Eryngium caucasicum* Trautv) populations (Javaherdeh, Kelardasht, Dohezar, Sehezar, Javaherdasht, and Eshkevar represented by black, green, red, blue, aqua, and blue‐violet color symbols, respectively) based on the biochemical traits and antioxidant activities by Euclidean distances

The Ward dendrogram/cluster analysis revealed two different clusters in terms of parameters measured, confirming high biochemical variability among the individual of populations (Figure 6). As shown, the individuals were classified into two major clusters. The first major cluster (I) comprised three individuals, forming two subclusters. Subcluster I‐A consisted of two individuals (Javaherdeh‐7 and Dohezar‐8), characterized by the maximum values of total flavonoid content and antioxidant activities (based on both DPPH and Fe^2+^chelating evaluations). However, subcluster I‐B consisted of only one individual (Dohezar‐10), characterized by the maximum amount of total phenolic content and antioxidant activity derived from the DPPH‐based method. In addition, the second cluster (II) was grouped into two subclusters (II‐A and II‐B). The majority of the individuals (43) were included in the II‐A cluster, distinguished by the highest essential oil content and moderate values of total phenolics and flavonoids and antioxidant activity. The rest of the14 individuals were grouped into the second subcluster (II‐B), characterized by the highest values of essential oil yield, rosmarinic acid and chicoric acid content (Figure 6). *Eryngium* L. species, as a rich and natural source of various chemicals/metabolites, are known for their importance in the field of nutritional and therapeutic purposes (Saroya et al., 2011; McClure et al., 2012).

**FIGURE 7:**
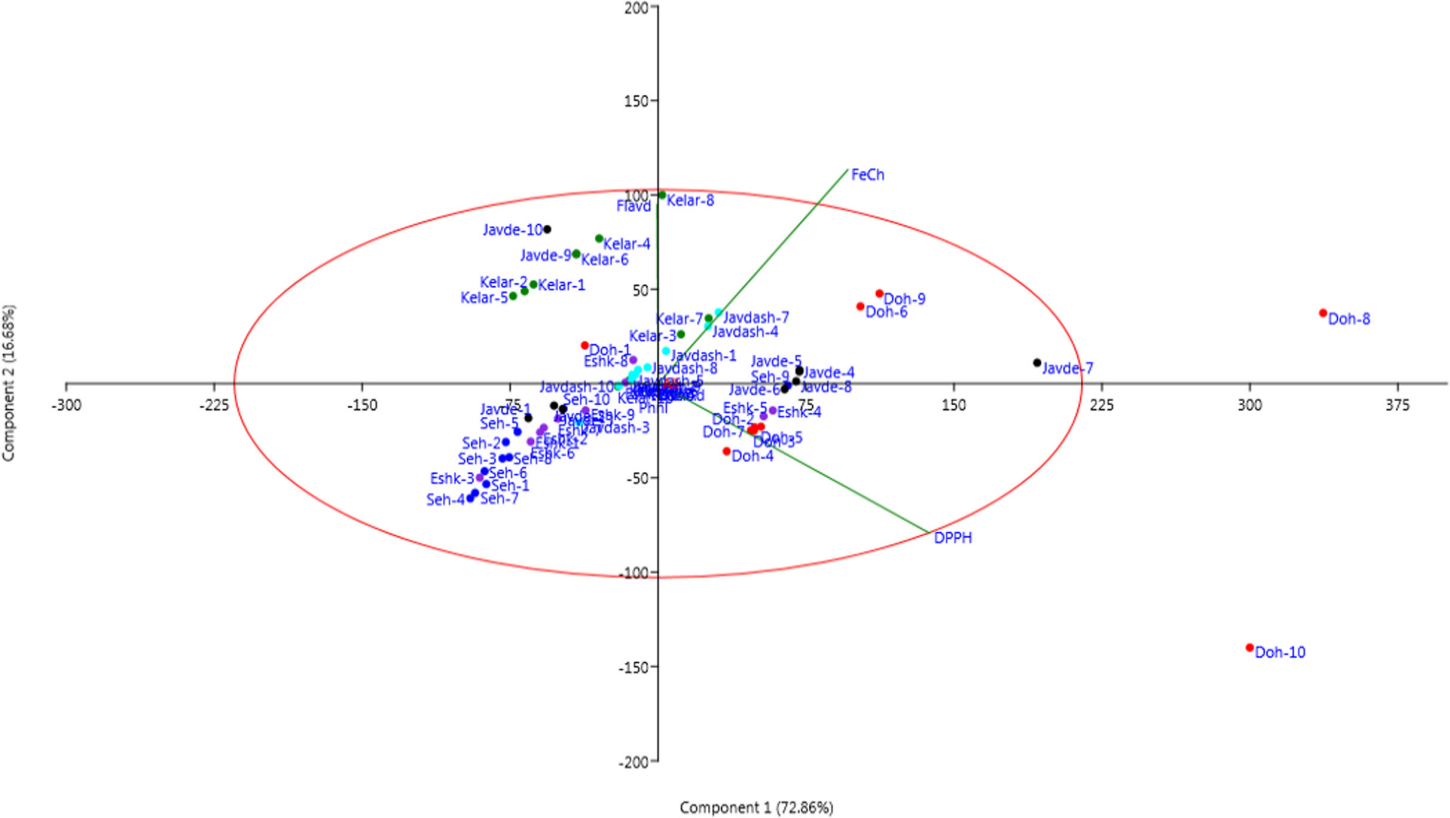
Two‐dimensional scatter plot for PC1 and PC2 based on the biochemical traits and antioxidant activities of the individuals (No.: 1–10) of eryngo (*Eryngium caucasicum* Trautv) populations (Javaherdeh, Kelardasht, Dohezar, Sehezar, Javaherdasht, and Eshkevar represented by black, green, red, blue, aqua, and blue‐violet color symbols, respectively)

The scatter plot (Figure 7) created using PC1 and PC2 showed variations among the individuals and separated the individuals into four groups. The scatter plot confirms that the results of biochemical traits and antioxidant evaluations are roughly similar to those of Ward dendrogram/cluster analysis.

**FIGURE 8:**
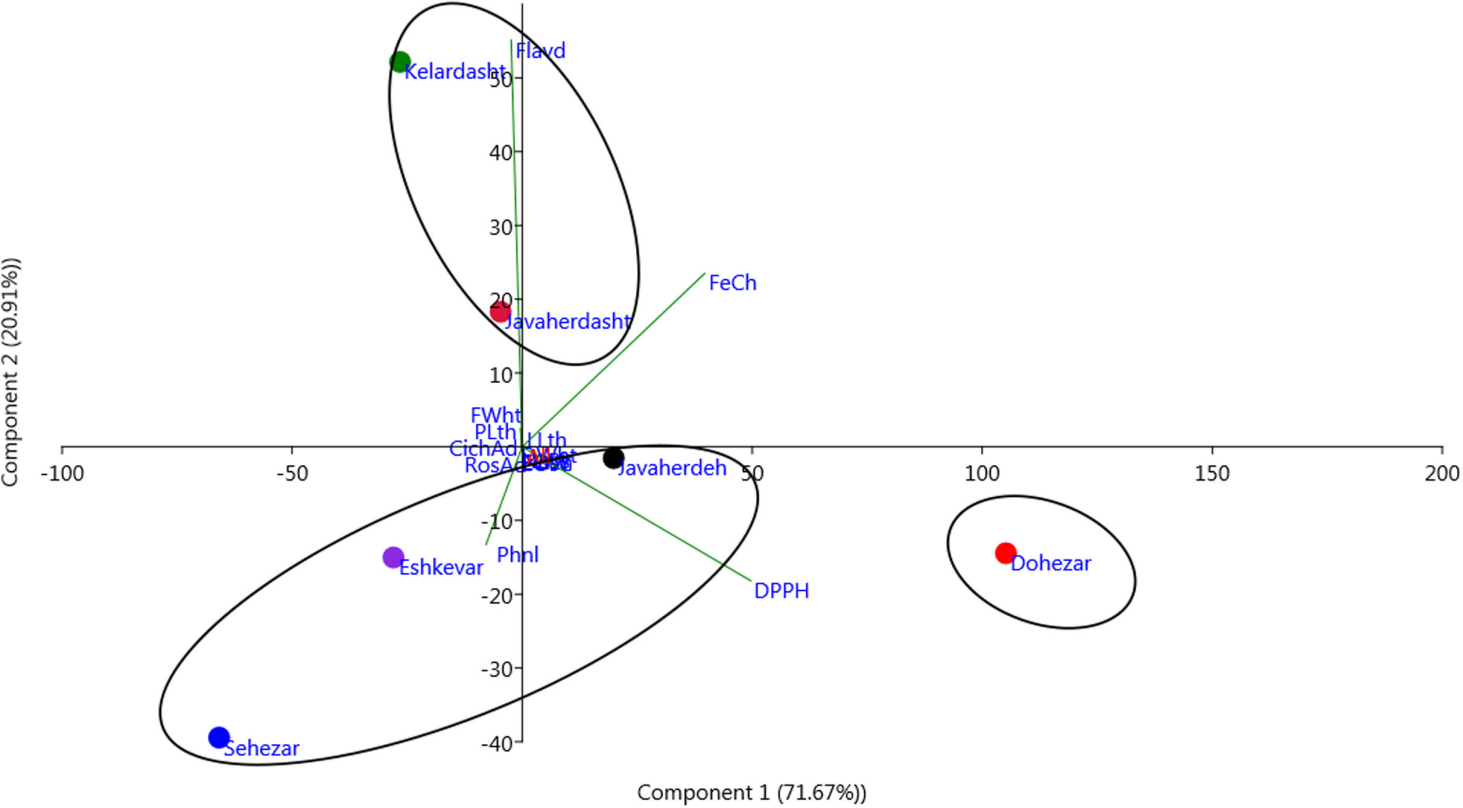
Biplot for the studied sites of eryngo (*Eryngium caucasicum* Trautv) populations including Javaherdeh, Kelardasht, Dohezar, Sehezar, Javaherdasht, and Eshkevar based on the morphological and biochemical characters and antioxidant activities

**FIGURE 9:**
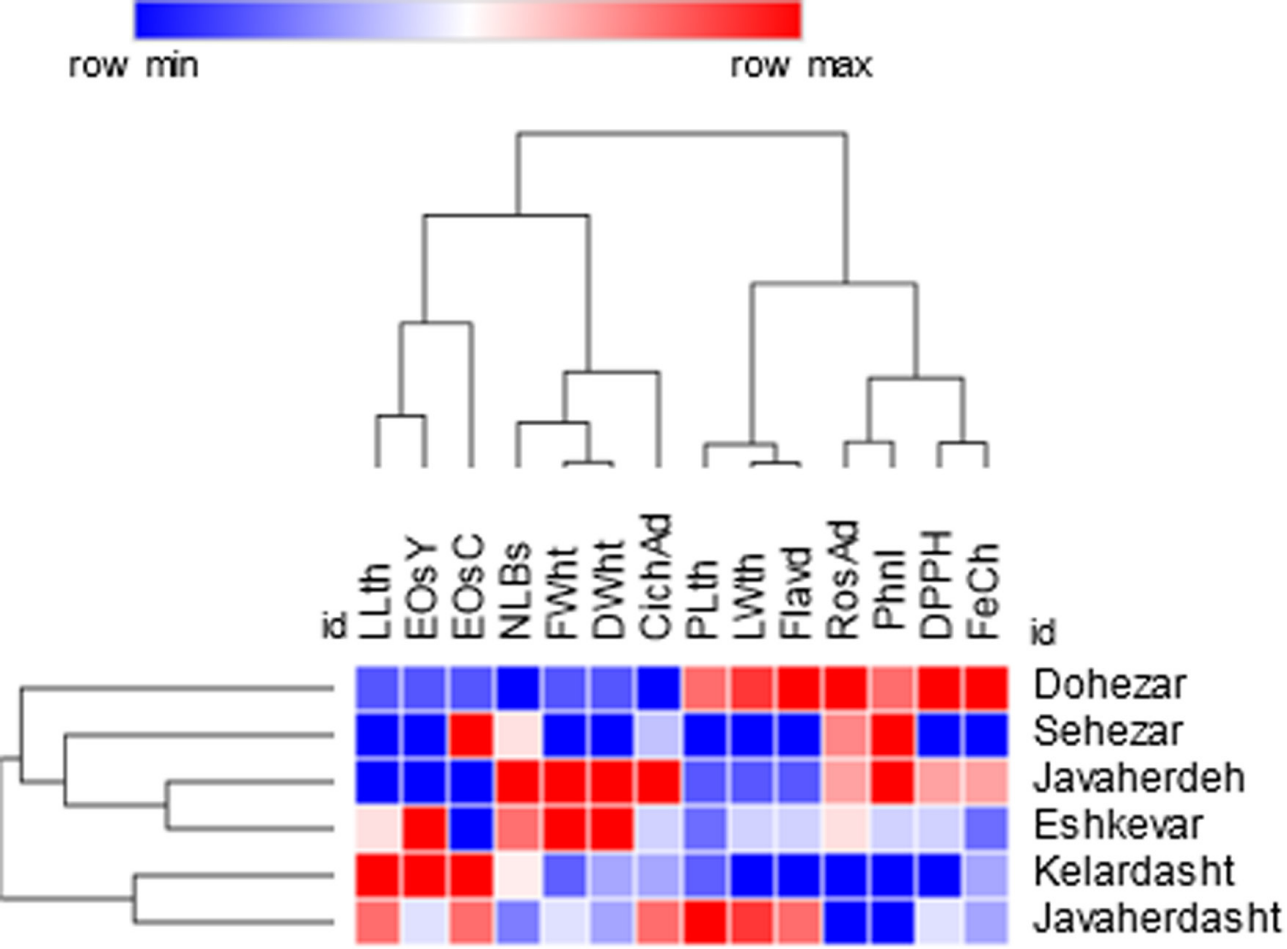
Hierarchical cluster analysis (HCA) for the studied populations of eryngo (*Eryngium caucasicum* Trautv) based on all morphological and biochemical characteristics and antioxidant activities. Red color represents a higher value than the mean for the specific trait in all the studied population, and while blue color shows a lower amount than the mean. For the explanation of traits’ abbreviation, please see Table 2

The population analysis of *E. caucasicum* based on all measured parameters is given in Figure 8. Biplot for the studied areas showed that the studied populations were classified into three groups. The “Dohezar” was placed in the first group and characterized by antioxidant activity with both DPPH and Fe^2+^ chelation methods, total flavonoids, and rosmarinic acid content. The second group consisted of three areas from Guilan and Mazandaran provinces and was divided into two subgroups. The first subgroup included “Sehezar,” while the second subgroup consisted of “Javaherdeh” and “Eskevar” locations (Figure 8). Similarly, the HCA showed clustering of studied variables to explore the similarity between observations and/or clusters based on the Euclidean distance coefficient and the average linkage technique (Figure 9). The result can be visualized using heat maps and dendrograms. The characters grouped together to react in a more similar way to the respective populations than those in other clusters. The diversity among populations may be related to natural hybridization/selection phenomenon, sexual reproduction, and human disturbances (Vilà et al., 2000; López‐Caamal and Tovar‐Sánchez 2014).

Based on overall analyses of all traits, a broad morphological and biochemical diversity was found among the populations, which could be useful in breeding programs and management of genetic resources. Furthermore, the previous studies conducted have shown the existence of genetic diversity and differentiation in *Eryngium* spp. from various countries/regions (Gaudeul et al., 2004; Minasiewicz et al., 2011; Ieviņa et al., 2019).

## 4 CONCLUSIONS

Eryngo (*Eryngium caucasicum* Trautv) is known for its importance in the field of herbal nutraceuticals. This species is rich in valuable bioactive compounds such as essential oils, phenolic acids, and flavonoids. The plant leaf traits concerning biomass are the most important characteristics to be considered in the agri‐food industry. The current study displayed a high diversity among and within populations of *E. caucasicum*. According to the multivariate analyses of the studied populations, the “Dohezar” was placed in the first group and characterized by antioxidant activity with DPPH and Fe^2+^ chelation methods, total flavonoids, and rosmarinic acid contents. The second group consisted of three areas from Guilan and Mazandaran provinces and was divided into two subgroups. The first subgroup included “Sehezar,” while the second subgroup consisted of “Javaherdeh” and “Eskevar” locations. Moreover, Based on the traits related to leaf dimensions and biomass such as leaf length, leaf width, petiole length, the number of leaves per plant, and shoot fresh and dry weights, as well as in terms of biochemical parameters related to antioxidant properties such as essential oils content and yield, total phenolics and flavonoids, rosmarinic acid and chicoric acid contents, and antioxidant activity, eight individuals plant including “Javaherdeh‐2”, “Javaherdeh‐7”, “Javaherdeh‐10”, “Dohezar‐4”, “Dohezar‐5”, “Dohezar‐6”, “Dohezar‐9”, and “Javaherdasht‐4” were superior, therefore, they could be valuable in breeding and field farming programs.

## CONFLICT OF INTEREST

The authors declare no conflict of interest.
